# Stress level and quality of life following pediatric cataract surgery

**DOI:** 10.5935/0004-2749.2021-0493

**Published:** 2023-03-08

**Authors:** Camila Ribeiro Koch, Marcelle Oliveira Parahyba, Adriana Nascimento Alves de Barros, Marcela Macedo Leite, Ângela Cristina Santana Souza, Joana Anjos Bastos

**Affiliations:** 1 Hospital Humberto Castro Lima, Salvador, BA, Brazil; 2 Universidade de São Paulo, São Paulo, SP, Brazil; 3 Hospital Santa Luzia, Salvador, BA, Brazil

**Keywords:** Cataract, Cataract extraction, Adverse childhood experiences, Quality of life, Child, Catarata, Extração de catarata, Experiências ad-versas da infância Qualidade de vida, Criança

## Abstract

**Purpose:**

To evaluate the quality of life and stress level related to visual function following pediatric cataract surgery in a Brazilian public hospital.

**Methods:**

This prospective study analyzed children aged 6-14 years old who underwent cataract surgery. The Childhood Stress Scale and Children’s Visual Function Questionnaire (CVFQ) were used to assess stress levels and quality of life, respectively. Both instruments were applied by two psychologists before and after the surgery. Eye examination was performed by two ophthalmologists. Preoperative and postoperative data were compared.

**Results:**

In total, 23 children (32 eyes) were enrolled in the study, of which 9 had bilateral cataracts. The average age group at the time of surgery was 9.65 ± 2.26 (6-14) years old. One month after the surgery, the spherical equivalent was -0.90 ± 1.66D, and the corrected distance visual acuity was 0.13 ± 0.10 (0-0.3) LogMAR in bilateral cases and 0.50 ± 0.39 (0-1.3) LogMAR in unilateral cases (p<0.01). According to the Childhood Stress Scale, 77.7% of the bilateral cases and 57.1% of the unilateral cases had stable stress levels, and 34.7% of the children improved their stress level. The analysis of the CVFQ was based on scores for general health, general vision health, competence, personality, and treatment. After cataract surgery, 78.2% of the patients had improved or maintained CVFQ scores in the general health domain; 82.6%, general vision health; 95.6%, competence; 56.5%, personality; and 78.2%, treatment.

**Conclusion:**

Pediatric cataract surgery improves the visual function and the quality of life even in patients undergoing surgical procedures, without increasing the stress levels.

## Abstract (pt)

**Objetivo:**

Avaliar a qualidade de vida e o nível de estresse relacionada à função visual após a cirurgia de catarata pediátrica em um hospital público brasileiro.

**Métodos:**

Estudo prospectivo em crianças de seis a 14 anos submetidas à cirurgia de catarata. A Escala de Stresse Infantil e o Questionário de Função Visual em Crianças foram usados para avaliar o nível de estresse e a qualidade de vida, respectivamente. Ambos os instrumentos foram aplicados por duas psicólogas antes e após a cirurgia. O exame oftalmológico foi realizado por dois oftalmologistas. Os dados coletados no pré e pós-operatório foram comparados.

**Resultados:**

Vinte e três crianças (32 olhos) foram incluídas no estudo, nove delas apresentavam catarata bilateral. A média de idade na cirurgia foi de 9,65±2,26 (6 a 14) anos. Um mês após a cirurgia, o equivalente esférico foi de -0,90 ± 1,66D e a acuidade visual corrigida a distância foi de 0,13 ± 0,10 (0-0,3) LogMAR em casos bilaterais e 0,50 ± 0,39 (0-1,3) LogMAR em casos unilaterais (p<0.01). De acordo com a Escala de Stresse Infantil, 77,7% dos casos de catarata bilaterais, e 57,1% dos casos unilaterais mantiveram o nível de estresse e 34,7% das crianças melhoraram o nível de estresse. A análise do Questionário de Função Visual em Crianças foi baseada em pontuações para saúde geral, saúde geral da visão, competência, personalidade e tratamento. Após a cirurgia de catarata, 78,2% dos pacientes melhoraram ou mantiveram o escore do Questionário de Função Visual em Crianças na saúde geral, 82,6% na saúde geral da visão, 95,6% na competência, 56,5% na personalidade e 78,2% no tratamento.

**Conclusão:**

A cirurgia de catarata pediátrica melhora a função visual e a qualidade de vida em pacientes submetidos a procedimento cirúrgico, sem aumentar o nível de estresse.

## INTRODUCTION

Stress conditions may impair physiological, cognitive^(1)^, and visual functions, further aggravating the situation^(2,3)^. Pediatric cataract remains one of the main causes of vision impairment and blindness worldwide^(4-6)^, and some pediatric cataracts require surgical treatment, which is also a psychosocial stressor. Therefore, stressful factors must be identified. Owing to occupational, economic, social, and psychological restrictions, cataract has negatively affect the quality of life^(7-10)^.

The Children Stress Scale (CSS) is a tool used in the assessment of child stress. It is applied on children with chronic diseases, blindness, and other comorbidities; however, no studies have assessed child stress levels after cataract surgery^(11,12)^. Children’s quality of life could be verified through the Children’s Visual Function Questionnaire (CVFQ), which is an instrument used to measure the effect of visual impairment on children and their families^(13)^. The CVFQ concerning pediatric cataract has demonstrated the worsening effects of visual impairment on children who have a history of unilateral as opposed to bilateral cataracts^(7)^. A study reported that senile cataract decreases vision quality, which affects the performance of daily-living activities causing dependence in mobility, reading, writing, and communication, but pediatric studies are scarce^(14)^.

Since the reduction in visual acuity (VA) implies a decreased quality of life and surgery is a trigger factor for stress, this study aimed to assess the quality of life and stress level of children who underwent cataract surgery, using the CVFQ and CSS.

## METHODS

### Study population

The prospective study involved children aged 6-14 years who underwent cataract surgery and were enrolled at the Humberto Castro Lima Hospital, in Salvador-Brazil, between January 2018 and January 2020. Informed consent was obtained from the parents/legal guardians of all participants before enrollment.

The study followed the tenets of the Declaration of Helsinki and was approved by the Medical Institutional Review Board along with the *Escola Bahiana de Medicina e Saúde Pública*.

The exclusion criteria were coexisting ocular disease, secondary cataracts following ocular trauma or uveitis, and previous ocular surgery. Patients with neurological impairments or who could not attend follow up appointments (because their residence is >250 km from the hospital) were also excluded. Children aged <6 years and >14 years were excluded because the CSS is applicable only for children aged 6-14 years.

### Patient data

Eligible patients underwent a complete eye examination at two different times: on the first appointment and 30 days following the cataract surgery. Two ophthalmologists were responsible for ophthalmological evaluation, which included VA (Snellen chart), cycloplegic refraction, slit-lamp biomicroscopy, applanation tonometry (Goldmann), and dilated fundus biomicroscopy. Lens opacities in mydriasis were graded using the slit-lamp^(15)^. The following variables were also collected: age, sex, corrected distance VA (CDVA) before and after surgery, total number of appointments, and number of days between the first appointment and the surgical procedure.

### CSS and QFVC

Two psychologists were responsible for the preoperative psychoprophylaxis of the children and administration of the assessment instruments before and after the surgery (after prescription and use of corrective lenses). The CSS and CVFQ were used. They are psychometric scales with questions, or statements, with different graduated answers, in a point Likert scale, with scores ranging from 0 to 4 (CSS) or to 5 (CVFQ) for each statement. The participants must respond according to the level of agreement or disagreement about the statement made, with 0 representing “not applicable” or “never,” 1 representing the “worst,” and 4 or 5 representing the “best” answer. The result is obtained through the sum of all points of each evaluated domain.

The CSS was validated to ascertain the stress level and type of children aged 6-14 years. The test contains 35 statements, regarding the child’s daily life, and each had five answer options with equal and ordinal intervals: “never,” 0 points; “a little,” 1 point; “sometimes,” 2 points; “often,” 3 points; and “always,” 4 points (maximum score). The scale is organized into four reactions and, according to the result, classifies the type of stress as follows: stress with physical reactions, stress with psychological reactions, stress with psychological reactions with a depressive component, and stress with psychophysiological reactions. Through the sum of the points, the type and level of child stress is obtained.

The CVFQ is a validated questionnaire that assesses the quality of life associated with visual function in children, adapted to the child’s competences and activities. It is available in two versions according to age range: <3 years and >3 years. This questionnaire must be completed by the parents or child’s legal guardians, regarding their perception of the child’s life quality, particularly related to their visual function. It contains 40 questions and statements, organized in five different domains: general health, visual function, competence, personality family influence, and treatment. The result is obtained by taking the average of each domain’s total score. The highest and lowest scores represent the “best” and the “worst” quality of life related to visual function, respectively. Family influence was part of the questionnaire answered by the parents, containing questions such as parents’ perception of the child feeling different in relation to others, how much they care about their child, time spent on treatments such as eye drops, tampons, use of glasses, among others.

### Psychoprophylaxis

During hospitalization, psychological preparation of children who will undergo a surgical procedure is routine. It must occur in an integrated manner, aiming for overall well-being. Psychology aims to diagnose and treat the psychological aspects surrounding the disease and its prevention. The preoperative preventive approach aims to help the patient confront specific disorders caused by cataracts and, in the case of this study, to relieve its symptoms in addition to emotionally prepare the patient for the surgery. Furthermore, it allows the detection of traumas produced by the surgical intervention, even preventing and reducing stressors during the treatment.

Psychoprophylaxis is performed by a psychologist during the preoperative assessment, always in the presence of the child’s legal guardian. The child was made to familiarize and introduced to the hospital staff, operating room, medicines, instruments, and all other materials that will be used during the surgical process playfully and understandably. At this time, it is possible to diagnose and treat previous traumas, answer questions, and explain the entire surgical procedure to the child and their legal guardian. This will help prevent psychological disorders, such as stress itself.

### Surgical procedure

Details of the surgical procedure of the children who underwent cataract surgery under general anesthesia were published^(16)^. Patients followed our usual postoperative schedule of ophthalmologic assessments (at postoperative days 1, 5 15^,^ and 30). In the last ophthalmic evaluation, refraction exam was performed, and prescription glasses were prescribed. Eye patches were also prescribed for those diagnosed with amblyopia, and these patients were followed by a pediatric strabismus ophthalmologist.

### Statistical analysis

Quantitative variables were presented by mean and standard deviation, whereas qualitative variables were expressed by absolute and relative frequencies. A p-value of <0.05 was considered significant. The one--sample t-test was used to analyze pre and postoperative data. The generalized estimating equation method was used to compare the means in patients who underwent surgery on one eye or both eyes. The IBM SPSS Statistics version 21 (IBM Corp., Armonk, NY, USA) was used for statistical analysis.

## RESULTS

A total of 29 children were included. Six selected children were excluded from the analysis for the following reasons: four did not follow up on their psychology appointments after surgery and two moved to another city. A total of 23 (32 eyes) were enrolled in the final analysis, of which nine had bilateral cataracts. Most of the cataracts were congenital or developmental. Only one patient with unilateral cataract was secondary to rhabdomyosarcoma treatment. The mean age at surgery was 9.65 ± 2.26 (6-14) years, and 14 (60.8%) were men. The most frequent cataract morphology was nuclear that was present in 10 eyes (31.3%), followed by subcapsular observed in 7 (21.9%) eyes. No intraoperative complications were observed. One patient with high myopia remained aphakic, and the others had intraocular lens in-the-bag implantation.

The time elapsed between the first consultation and the surgical procedure was <30 days in 39.1%; between 1 and 2 months in 34.8%; 2 months in 17.4%; and 4 or 5 months in 4.3% of the children. The number of total appointments before and after surgery was 7.43 ± 2.10 (3-12).

### VA and refraction outcomes

The mean preoperative CDVA was 0.93 ± 0.39 (0.2-1.3) LogMAR. VA improvement was observed after surgery (p<0.01). Of the 23 patients, 19 presented a considerable VA improvement and 5 a slight VA improvement. No patient had worsened VA when preoperative and postoperative values were compared. One month after surgery, the mean CDVA in bilateral cases was 0.13 ± 0.10 (0-0.3) LogMAR and 0.50 ± 0.39 (0-1.3) LogMAR in unilateral cases (p<0.01). The mean CDVA was 0.29 ± 0.32 (0-1.3) LogMAR, and the mean spherical equivalent (SE) was -0.90 ± 1.66D (2.87 to -3.25).

### Main outcomes

According to the CSS, among the nine patients who underwent bilateral cataract surgery, six children did not show signs of stress, two were in the alert phase of stress, and one remained in the alert phase. Regarding 14 patients with unilateral cataract, seven did not show signs of stress, two were in the alert phase, and three had attenuated stress levels. In this group, two patients had no signs of stress, whereas one child had worsened, downgrading from the resistance phase to a more severe alert phase. Details information is provided in the supplementary materials. The table separated the results by characteristics: physical, psychological, psychological with depressive components, and psychophysiological. The numbers represent the sum, defined in the test. Table 1 summarizes the outcomes according to the reactions of the patients.

**Table 1:** Outcomes of reactions according to the Childhood Stress Scale

Child	Physical		Psychological		Psychological with depressive components		Psychophysiological		Total		Stress stages	
	Pre	Post	Pre	Post	Pre	Post	Pre	Post	Pre	Post	Pre	Post
1	7	2	11	7	1	10	9	1	28	20	N	N
2	2	16	11	15	7	10	9	10	29	51	N	A
3	1	1	0	0	0	0	5	5	6	6	N	N
4	8	8	8	6	6	4	4	9	26	27	N	N
5	12	13	6	16	2	9	10	7	30	45	N	A
6	9	1	6	2	0	0	15	13	30	16	N	N
7	3	4	4	8	4	1	3	6	14	19	N	N
8	4	4	1	1	1	1	4	4	10	10	N	N
9	4	10	13	16	12	0	11	8	40	34	A	N
10	11	5	8	1	8	0	8	3	35	9	N	N
11	5	9	17	21	6	14	7	15	35	59	N	A
12	9	16	11	16	7	16	13	10	40	58	A	A
13	11	8	15	11	2	1	8	4	36	24	N	N
14	9	11	9	12	0	8	16	15	34	46	N	A
15	3	4	13	4	5	5	9	3	30	16	N	N
16	9	6	15	16	1	2	4	7	29	31	N	N
17	10	4	11	10	2	0	6	4	29	18	N	N
18	13	9	26	29	16	7	12	10	67	55	R	A
19	10	13	14	16	8	10	10	12	42	51	A	A
20	12	4	23	11	9	20	10	7	54	42	A	A
21	12	0	23	12	0	0	12	16	47	28	A	N
22	9	8	6	4	2	2	8	1	25	15	N	N
23	2	6	9	9	2	3	11	11	24	29	N	N

N= normal; Stress stages: A= alert; R= resistance; NE= near exhaustion; E= exhaustion. Pre= preoperative; Post= postoperative.

Gray squares signalize stress.

**Figure 1:**
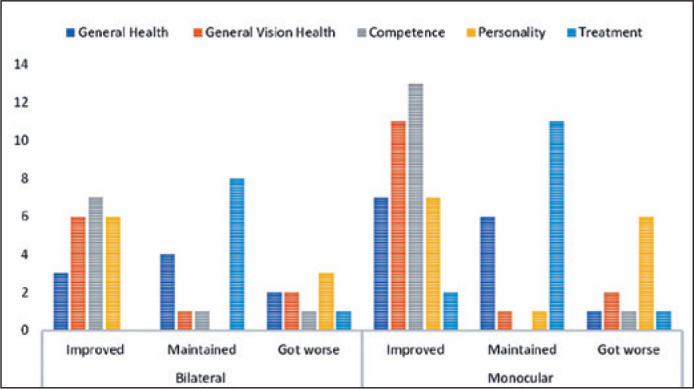
Outcomes in the subscale scores in the Children’s Visual Function Questionnaire according to the laterality.

Figure 1 shows the results in the subscale scores according to the laterality in the CVFQ. Most of the patients had improved or maintained scores in all domains; however, variations were noted in the analyzed domains according to laterality. Regarding bilateral cases, four children had maintained general health scores, whereas three had improved and two worsened scores. Six improved, two had worsened, and one had maintained general vision. Seven had improved, one was kept stable, and one had worsened scores, within their respective competence domain. Six had improved and three had worsened scores in the personality domain. Eight had maintained stable and one patient got worse scores in the personality domain. Concerning patients with monocular cataract, seven had improved, six maintained stable, and one had deteriorated overall health. In the general vision health domain, 11 had improved, two had worsened, and one had plateaued scores. Thirteen had improved and one got worse scores in the competence domain. In the personality domain, seven had improved, six got worse, and one had kept stable scores. In the treatment domain, 11 had plateaued, two had improved, and one got worsened scores.

In patients who had two surgeries, seven considered worsening status. In addition to two surgeries and two postoperative periods, the time spent by those responsible for care is greater, which also suggests a greater effect. This is also observed in patients who underwent only one surgery, which suggests that the influence of family is attributed to postoperative care and different conditions, such as the use of glasses after surgery, which has repercussions on the comments made by third parties, whether going to a pharmacy, in the family circle, in addition to using eye drops, which did not happen even before the surgery. The change in the routine in this component of the questionnaire indicates this “worsening” in the family influence, as it increases care and attention and is not related to the patient’s field of vision.

Of the nine children who underwent two surgeries, no sign of stress was found in most of them (66.6%). Of the nine bilateral cataract cases included in this study, only two children showed worsening of stress levels, despite the improvement in VA. The others had maintained stable stress levels. Regarding the 14 children who had only one surgery, half of them showed no signs of stress. Three of them improved, three were no longer stressed, and one had an improved level of resistance and upgraded to the alert phase. Other patients had maintained stable stress levels. Regarding patients who had improved stress levels: two did not have significantly improved VA, whereas four of them did.

## DISCUSSION

Some pediatric cataracts require surgical intervention to improve VA. Pathology-related limitations experienced by a child can affect their quality of life. Hospitalization causes stress particularly in patients undergoing surgery^(17)^. This study evaluated the visual function and stress levels of these children who underwent surgery, CVFQ was used to assess patients’ perception of the quality of life, whereas the CSS was used to evaluate stress levels^(13)^. According to our outcomes, cataract surgery could improve visual function and quality of life, reducing or keeping the levels of stress stable.

The instruments were applied after psychoprophylaxis. It can explain the results found in the CSS questionnaire, which shows that stress levels of most patients did not worsen, even after surgical procedure. Children who are aware of the importance of surgery for their well-being are calmer and more cooperative in relation to post-surgical procedures^(18)^. Preparing the patient appropriately is the most effective way to reduce stress, even more effective than the presence of the mother. However, the characteristics of each child, age, sex, education, disease type, surgery type, health condition, previous surgical experience, family, and sociocultural integration must be considered.

Stressors are classified as internal and external. Internal stressors result from the way the individual faces situations and reacts to them, whereas external stressors represent any event in the external environment that requires adaptation. Psychoprophylaxis in elective pediatric surgery could attenuate anxiety in children, clarify doubts, and contemplate internal stressors. In this study, external events, such as adaptation to the hospital environment and integration with the team are included in the preoperative interview. Health issues, surgical intervention awareness, surgical event itself, and hospitalization can be defined as stressors. Moreover, several ophthalmological assessments such as school absences, use of eye drops, use of glasses, and low economic condition for the treatment were stressors identified in this study^(17)^.

The “quality of life” can be a social representation created from subjective parameters (well-being, happiness, love, pleasure, and personal fulfillment) and goals, whose references are the satisfaction of the basic needs and needs created by the degree of economic and social development of a society. The CVFQ is an instrument to measure the effect of visual impairment on children and their families^(13)^. The pathology-related limitations experienced by children can mean changes in their quality of life. Specific situations must be considered, such as other pathologies, as in patient 19 in this study, who developed cataracts after completing the rhabdomyosarcoma treatment, which, despite the significant gain in VA, has maintained stable stress levels.

In this study, we expected to find an improvement in VA after surgery and a better quality of life, especially in children with bilateral cataracts because of a lower risk of amblyopia. However, the results brought other perspectives. The results in the CVFQ varied depending on the domain analyzed. In relation to general health, three patients had worsened states, although they showed an improvement in VA. In the “general health of vision” domain, four children reported worsening stress levels, two reported no change, and only one patient reported improvement, despite all having improved VA. Regarding competence, two patients had worsened, with a slight improvement in VA, and the others had improved or maintained their assessment results.

Studies using this instrument in pediatric cataract demonstrated that the effects of visual impairment on the family domain are worse in families of children with unilateral cataracts compared to those with bilateral ones and that the competence domain is reduced in children with bilateral cataracts compared with healthy controls^(19)^. Regarding the family influence in this study, seven patients worsened states, and of these, only three did not show considerable improvement in VA, and the others had improved or maintained levels. Regarding personality, 11 showed worsening states, and only three did not show significant improvement in VA. Finally, for treatment, there was a worsening in adherence in only two patients.

Two results are noteworthy in the family influence domain, in which seven patients had negative results. Another important aspect was personality, in which worsening states were noted in 11 of the children. This result drew attention and could be affected by the criteria considered, such as going out with friends, family, playing games, and watching TV. It is precisely due to the non-adaptation to wearing glasses, for example, and perhaps comments that could have retracted the child; thus, they would not want to go out and interact. Another possible reason was the time needed for the patient to visually readapt, which made the guardians overprotective, not allowing their child to engage in more intense physical activities due to fear of potential injuries. The children did not have access to or develop a taste for reading, for example, something that became even more difficult than before. The limitation of this study was the sample size.

In summary, it is important to prepare the child--patient for the surgical event, including its pre and postoperative stages, through psychoprophylaxis. Therefore, internal and external stressors in the child’s and parents’ life should be identified. This study showed that pediatric cataract surgery improves the quality of life associated with visual function in addition to optimizing or maintaining stable stress levels after surgery. Improving VA and better assessing the social and emotional aspects of the patient who needs cataract surgery, and their family members are essential elements in this process. Future surveys may include other age another age group, other assessment intruments to acess level of stress and quality of life and longer follow-up.

## References

[1] Kumar A, Das S, Chauhan S, Kiran U, Satapathy S. (2019). Perioperative anxiety and stress in children undergoing
congenital cardiac surgery and their parents: effect of brief intervention-a
randomized control trial. J Cardiothorac Vasc Anesth.

[2] Kempen GI, Ballemans J, Ranchor AV, van Rens GH, Zijlstra GA. (2012). The impact of low vision on activities of daily living, symptoms
of depression, feelings of anxiety and social support in community--living
older adults seeking vision rehabilitation services. Qual Life Res.

[3] Fiszson Herzberg V (2014). [Pediatric surgical psychoprophylaxis: a benefit in the quality
of care of our youngest patients]. Rev Calid Asist.

[4] Lim ME, Buckley EG, Prakalapakorn SG. (2017). Update on congenital cataract surgery management. Curr Opin Ophthalmol.

[5] Thouvenin D. (2011). [Management of infantile cataracts: surgical technics and choices
in lens implantation]. J Fr Ophtalmol.

[6] Repka MX. (2016). Visual Rehabilitation in Pediatric Aphakia. Dev Oph-thalmol.

[7] Birch EE, Cheng CS, Felius J. (2007). Validity and reliability of the Children’s Visual Function
Questionnaire (CVFQ). J AAPOS.

[8] Flaxman SR, Bourne RR, Resnikoff S, Ackland P, Braithwaite T, Cicinelli MV, Vision Loss Expert Group of the Global Burden of Disease
Study (2017). Global causes of blindness and distance vision impairment
1990-2020: a systematic review and meta-analysis. Lancet Glob Health.

[9] Tartarella MB, Britez-Colombi GF, Milhomem S, Lopes MC, Fortes JB. (2014). Pediatric cataracts: clinical aspects, frequency of strabismus
and chronological, etiological, and morphological features. Arq Bras Oftalmol.

[10] Rezende MS, De Biagi Souza S, Dib O, Branzoni E, Ribeiro LE. (2008). Abordagem da catarata congênita: análise de
série de casos approach to congenital cataract: case series
analysis. Rev Bras Oftalmol.

[11] Menk TA, Inácio M, Macedo DB, Bessa DS, Latronico AC, Mendonca BB (2017). Assessment of stress levels in girls with central precocious
puberty before and during long-acting gonadotropin--releasing hormone
agonist treatment: a pilot study. J Pediatr Endocrinol Metab.

[12] Serra-Negra JM, Paiva SM, Flores-Mendoza CE, Ramos-Jorge ML, Pordeus IA. (2012). Association among stress, personality traits, and sleep bruxism
in children. Pediatr Dent.

[13] Casslén B, Jugård Y, Taha Najim R, Odersjö M, Topa A, Andersson Grönlund M. (2020). Visual function and quality of life in children and adolescents
with anophthalmia and microphthalmia treated with ocular
prosthesis. Acta Ophthalmol.

[14] Skalicky SE, Martin KR, Fenwick E, Crowston JG, Goldberg I, McCluskey P. (2015). Cataract and quality of life in patients with
glaucoma. Clin Exp Ophthalmol.

[15] Amaya L, Taylor D, Russell-Eggitt I, Nischal KK, Lengyel D. (2003). The morphology and natural history of childhood
cataracts. Surv Ophthalmol.

[16] Koch CR, Santhiago MR, Jorge PA, Sena P, Kara-Júnior N. (2020). Posterior capsule opacification after cataract surgery in
children over five years of age with square-edge hydrophobic versus
hydrophilic acrylic intraocular lenses: a prospective randomized
study. Clinics (São Paulo).

[17] Self JE, Taylor R, Solebo AL, Biswas S, Parulekar M, Dev Borman A (2020). Cataract management in children: a review of the literature and
current practice across five large UK centres. Eye (Lond).

[18] Silva RD, Austregésilo SC, Ithamar L, Lima LS. (2017). Therapeutic play to prepare children for invasive procedures: a
systematic review. J Pediatr (Rio J).

[19] Lopes MC, Salomão SR, Berezovsky A, Tartarella MB. (2009). [Assessing vision-related quality of life in children with
bilateral congenital cataracts]. Arq Bras Oftalmol.

